# Endodontic management of mandibular canine with two roots and two canals: a rare case report

**DOI:** 10.1186/s13104-018-3226-8

**Published:** 2018-02-08

**Authors:** Deepak Kumar Roy, Stephen Cohen, Varun Pratap Singh, Vinay Marla, Siddharth Ghimire

**Affiliations:** 1 Department of Conservative Dentistry and Endodontics, Kathmandu Medical College, Kathmandu, Nepal; 2 Arthur A. Dugoni School of Dentistry, University of the Pacific, San Francisco, California USA; 3 AL Ain Dental Center, SEHA Ambulatory Health Care Services, Alain, UAE; 4 Department of Oral Pathology, Kathmandu University of Medical Science, Dhulikhel, Nepal

**Keywords:** Mandibular canine, Two canals, Two roots

## Abstract

**Background:**

In general, mandibular canines have a single root and a single canal. The occurrence of two roots and two canals is a rare entity ranging from 1 to 5%. The anatomy of root canal morphology plays a decisive role in determining the conditions under which endodontic treatment can be performed effectively. Successful endodontic treatment comprises proper diagnosis, meticulous cleaning and shaping followed by three dimensional obturation. Failure to do so may lead to postoperative diseases, pain and further complications. This paper reports successful management of a mandibular canine with two roots and two canals.

**Case presentation:**

45-year-old Nepalese women with a non-significant medical history presented with a chief complaint of pain in a lower left front tooth. The pain disturbed her sleep and lingered for several minutes even after removal of a thermal stimulus. Clinical examination and testing revealed generalized severe attrition with tenderness to percussion in the mandibular left canine. Electric pulp test for all the mandibular anteriors revealed almost no response in the mandibular left canine. Intraoral periapical radiographs in different angulations were taken which revealed two roots and two canals. A clinical diagnosis of chronic irreversible pulpitis with symptomatic apical periodontitis was made and root canal therapy was performed following the standard protocols.

**Conclusion:**

Although the prevalence of two roots and two canals in mandibular canine is very low, the clinician should always be mindful of variations in the number of roots and canals for proper management of such cases.

## Background

Root canal morphology plays a decisive role in determining the conditions under which the endodontic treatment can be performed effectively [1]. Successful endodontic treatment comprises proper diagnosis, meticulous cleaning and shaping and three dimensional obturation. Failure to do so may lead to postoperative diseases, pain and further complications [2]. Therefore, the clinician should be aware of any anatomical variations which may alter the prognosis for root canal therapy. One of the most common reasons for failure of endodontic treatment is a missed canal due to lack of knowledge on anatomical variations [3].

Generally, mandibular canines contain a single root and canal [1]. The occurrence of two roots and two canals is a rare entity ranging from 1 to 5% [1, 4]. Although the prevalence of two roots and two canals in a mandibular canine is very low [1, 2, 5, 6], the clinician should be mindful of variations in the number of roots and canals for proper case management. Unpredictable findings in the root canal morphology of mandibular canines have a great impact in endodontic treatment [6]. This paper reports successful management of a mandibular canine having two roots and two canals.

## Case presentation

**Fig. 1:**
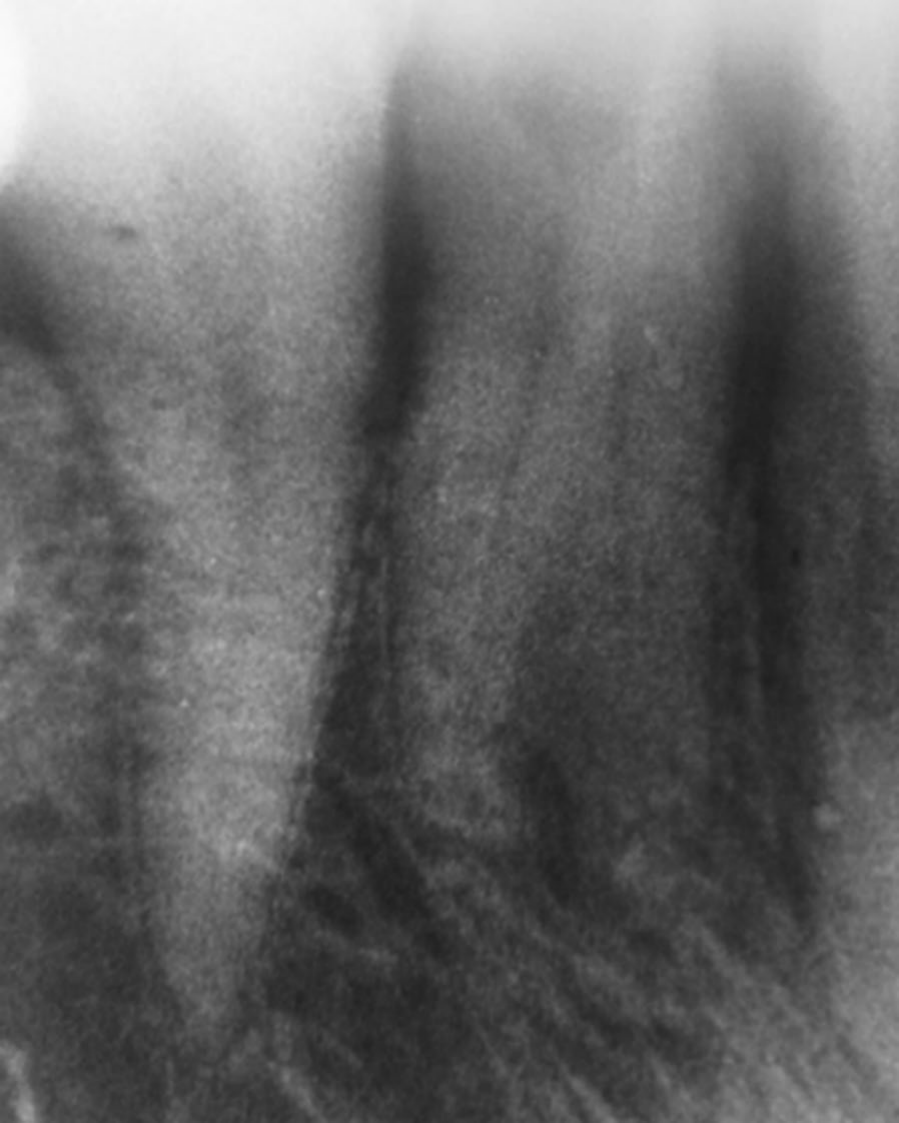
Diagnostic radiograph showing two roots

A 45 -year-old Nepalese women with a non-significant medical history reported to the Department of Conservative Dentistry and Endodontics, at B. P. Koirala Institute of Health Sciences, Dharan, Nepal with the chief complaint of pain in a lower left front tooth. The pain disturbed her sleep and lingered for several minutes even after removal of a thermal stimulus. Clinical examination revealed generalized severe attrition of her teeth with tenderness to percussion on the mandibular left canine. Electric pulp testing was performed for all the mandibular anteriors. Almost no response to the electric pulp test was observed in mandibular left canine. Intraoral periapical radiographs in different horizontal angulations were taken which revealed two roots (Fig. 1). A clinical diagnosis of chronic irreversible pulpitis with symptomatic apical periodontitis was made and root canal therapy was recommended.

**Fig. 2:**
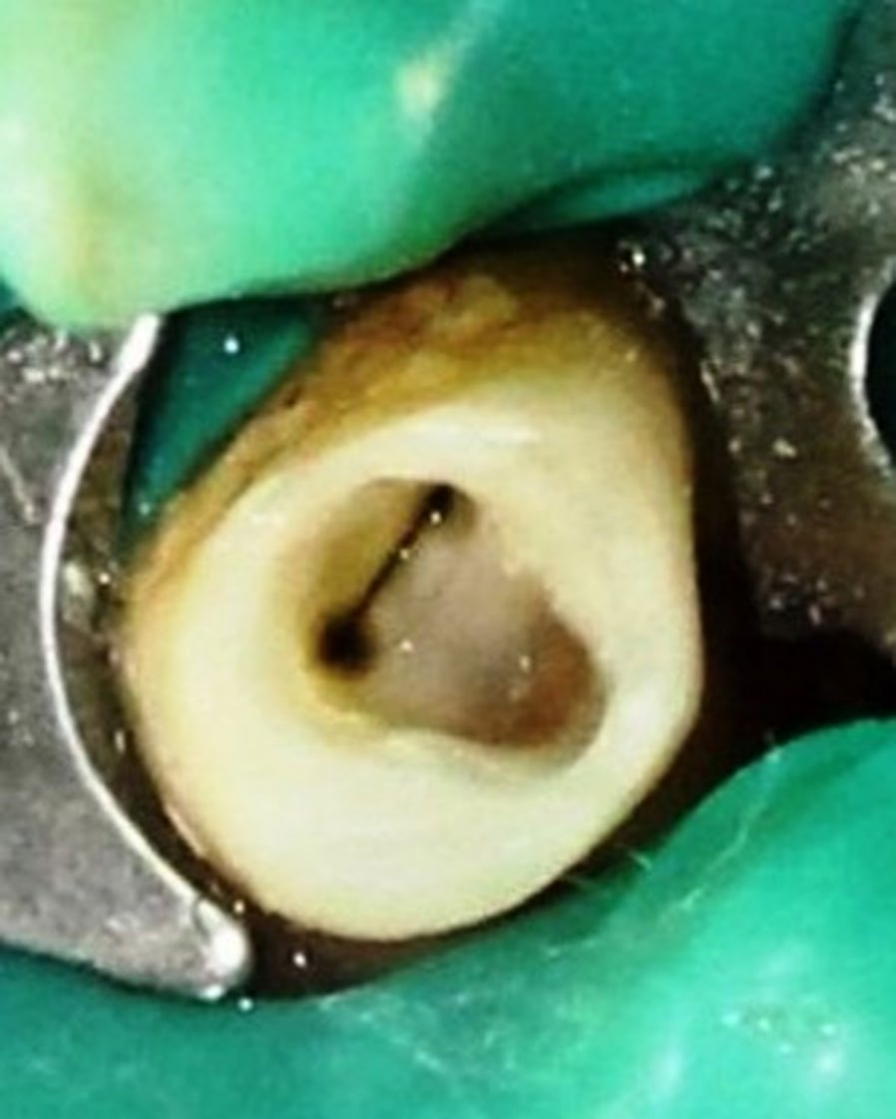
Two canals seen after access opening

Local anesthesia was administered and the entire procedure was performed under a rubber dam. The access opening was prepared with an endo access # 1 round diamond bur and endo-Z tapered safe-end bur (Fig. 2).

**Fig. 3:**
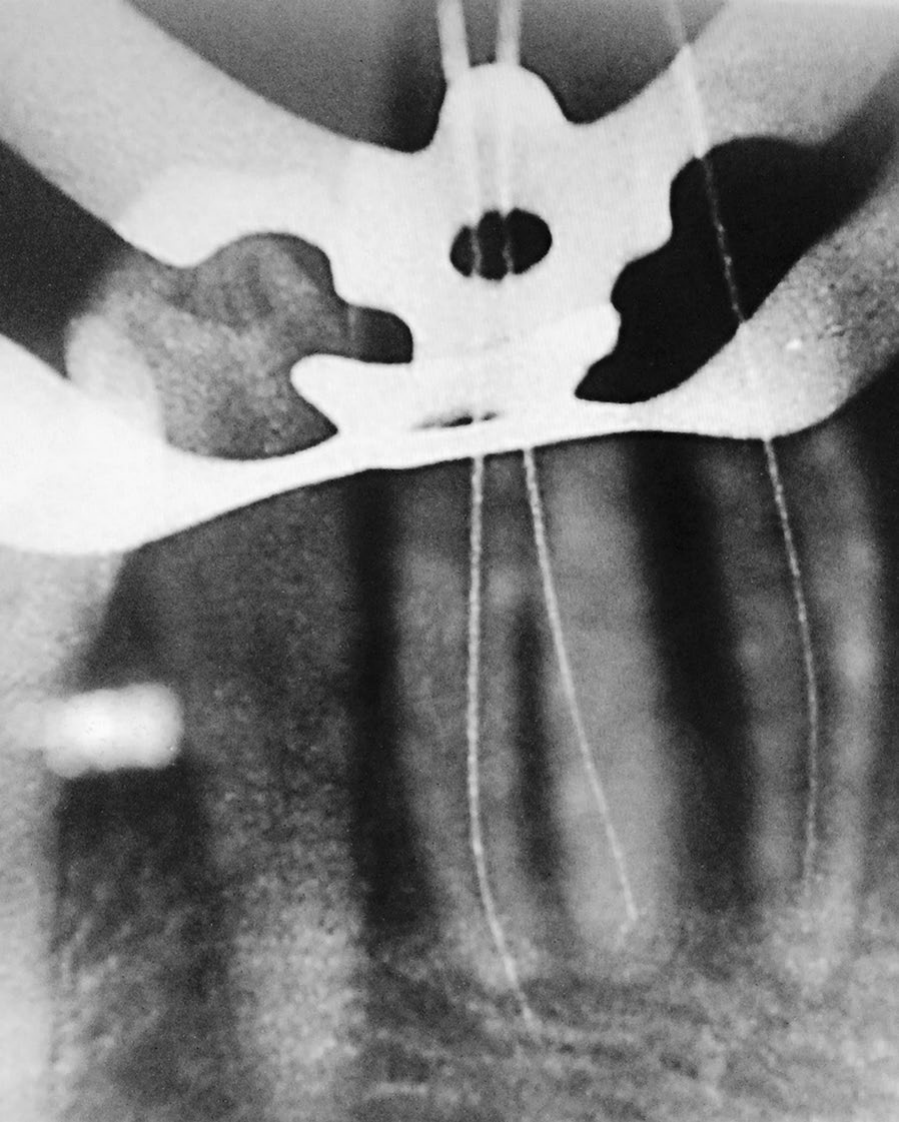
Test file radiograph to determine the working length

**Fig. 4:**
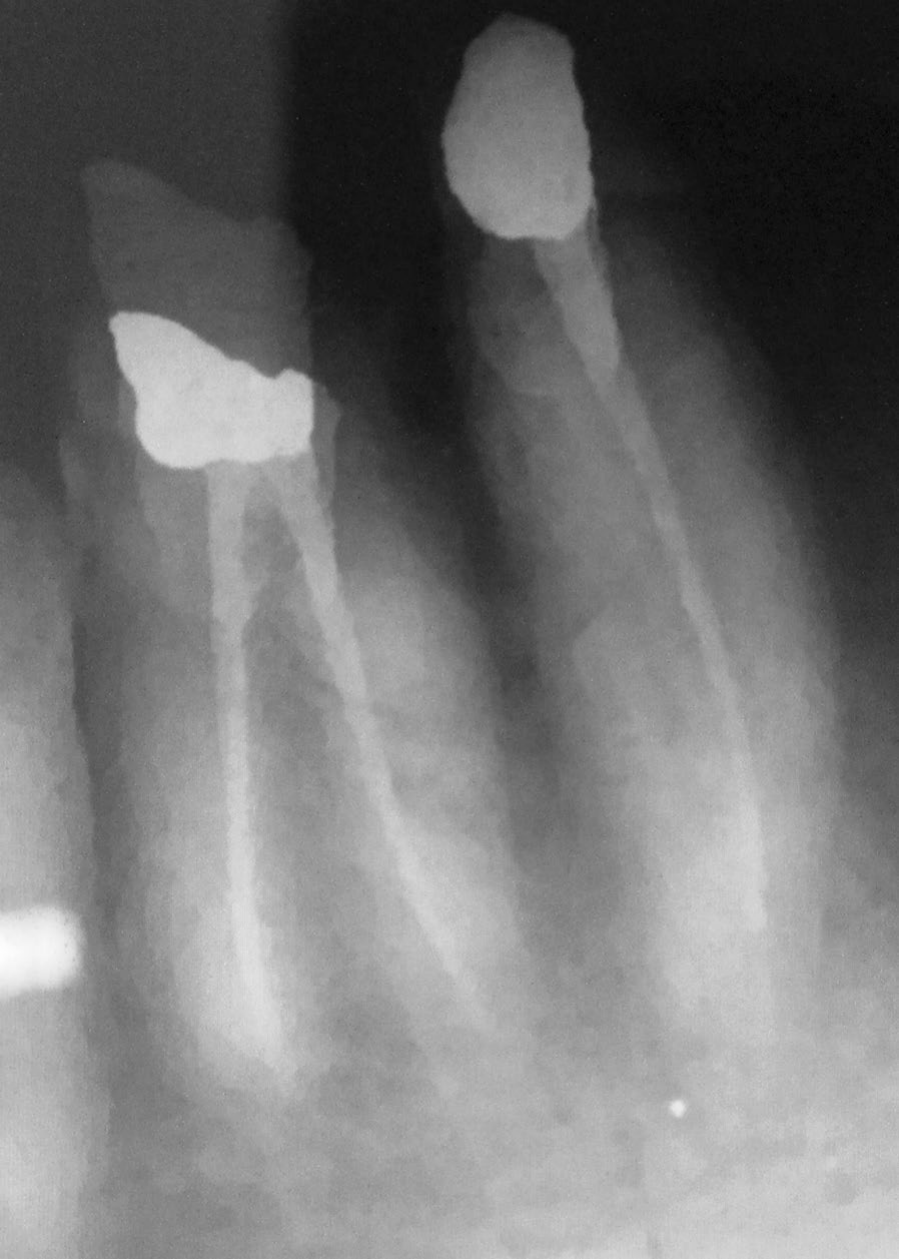
Radiograph showing obturated both canals

After reaching the pulp chamber we found that there were two canal orifices situated labial and lingual. This finding was different from the usual single orifice located in the center of the crown/root. Hence, size Nos. 6, 8 and 10 K files were used for creating the glide path and No. 15 K file was used for determining the working lengths (Fig. 3). The radiographs were exposed in two different angulations to confirm the presence of two canals. The cervical and middle thirds were prepared with a hand file with master apical filing up to No. 35. After each file, the canals were irrigated with 5.25% sodium hypochlorite and 17% ethylene diaminetetraacetic acid (EDTA). The root canals were dried with paper points and obturated with gutta percha cones and AH-Plus Sealer using the lateral compaction technique (Fig. 4). The access opening was sealed with a glass ionomer cement as a base and completed with a bonded composite as a permanent restoration.

## Discussion and conclusion

Morphologically, mandibular canines are usually mono-radicular [1]. The general anatomy with single root and canal is not always same for every mandibular canine. Some atypical finding like, two root canals, one or two roots with three canals; as well as two roots and two canals have been reported [2, 5, 7–9]. The complex anatomy of the mandibular canine should be thoroughly understood for proper management and a better prognosis [8, 10]. The clinician must be mindful of variations in root canal anatomy. Mindfulness should be taken from the beginning of the treatment until its completion because endodontic treatment becomes technically difficult when unexpected complexity is found in root canal [7]. This case reports successful management of a mandibular canine with two roots and two canals.

The study on root canal morphology of mandibular canine and the prevalence of the number of roots and canals has not yet been done in Nepal. This is the first report of such a case from Nepal. Different authors have put forward their view on the roots and canal morphology of mandibular canine [1–5]. Vertucci [11] in a study observed 18% of canines having two canals. Similarly, Green D [12] observed two canals in a single rooted canine in 13 out of 100 teeth examined. It is very unusual to find two roots and two canals in a mandibular canine. Arcangelo et al. [13] presented two cases of two roots and two canals. Laurichesse et al. [9] reported 2% of mandibular canine having one root and two canals and only 1% having two roots and two canals. Likewise, Pecora et al. [6] in a study on 830 teeth found that 1.2% had two canals with two orifices in a single root and 1.7% had two separate roots. Bakianian et al. [14] reported 12% of cases having two canals. Kaffe et al. [7] examined 400 mandibular canines; and Pineda and Kuttler [10] studied 187 teeth radiographically and concluded that the number of two canals were observed in 13 and 18.5% respectively. Mandibular canines with very unusual presentation like two roots and three canals; and three root canals have been reported by Heling et al. [15] and Orguneser and Kartal [16] respectively.

Intraoral periapical radiographs are essential for identifying the internal anatomy of the tooth. Radiographs taken in different horizontal angulations helps in better visualization of the canals. Often a single radiograph made at the vertical and horizontal projection dose not displays all the roots and canals. In order to gain the access in confirming the number of roots and canals, tube shift technique/buccal object rule/Clark’s Rule also called as SLOB (same Lingual Opposite Buccal), technique can be used [17]. In the present case, we projected two X-rays, one at − 20° and the second projection was altered 10°–15° mesially. Despite the use of different techniques for taking an intraoral periapical radiograph, there may be a chance of superimposition and distortion of the image [17]. The images may get foreshortened or elongated when an angle is altered while taking a radiograph. In order to overcome these shortcomings, cone beam computed tomography (CBCT) can be used for those cases to accurately determine the number of roots, curvatures, and bifurcations in both sagittal and axial planes [10, 18].

Because the location of canals is different from normal variants, care should be taken while preparing the access cavity because in appropriate angulations of the bur may lead to iatrogenic mishaps. Apart from endodontic concern in managing mandibular canines with two roots and two canals, canines hold a great importance in the field of orthodontics for tooth movement [19–21].

Considering the facts from different studies it can be expected that the presence of root canal variations in mandibular canine is an uncommon finding, but the clinician should always be aware about anatomical diversities before endodontically treating any tooth. This case highlights the importance of meticulous exploration of the roots and canals during endodontic treatment of mandibular canines. When in doubt additional diagnostic techniques like tube shift techniques and cone beam computed tomography (CBCT) can be utilized for accurate identification of extra canals. Thorough knowledge of anatomical variations of root canal morphology is always essential for effective endodontic management of cases.

## Abbreviations

EDTA: ethylene diaminetetraacetic acid
CBCT: cone beam computed tomography
